# Interviewing Suspects with Avatars: Avatars Are More Effective When Perceived as Human

**DOI:** 10.3389/fpsyg.2016.00545

**Published:** 2016-04-21

**Authors:** Sabine Ströfer, Elze G. Ufkes, Merijn Bruijnes, Ellen Giebels, Matthijs L. Noordzij

**Affiliations:** ^1^Department of Psychology of Conflict, Risk and Safety, University of TwenteEnschede, Netherlands; ^2^Department of Human Media Interaction, University of TwenteEnschede, Netherlands; ^3^Department of Cognitive Psychology and Ergonomics, University of TwenteEnschede, Netherlands

**Keywords:** deception, lying, virtual avatar, electrodermal activity, operation, suspect interview

## Abstract

It has been consistently demonstrated that deceivers generally can be discriminated from truth tellers by monitoring an increase in their physiological response. But is this still the case when deceivers interact with a virtual avatar? The present research investigated whether the mere “belief” that the virtual avatar is computer or human operated forms a crucial factor for eliciting physiological cues to deception. Participants were interviewed about a transgression they had been seduced to commit, by a human-like virtual avatar. In a between-subject design, participants either deceived or told the truth about this transgression. During the interviews, we measured the physiological responses assessing participants' electrodermal activity (EDA). In line with our hypothesis, EDA differences between deceivers and truth tellers only were significant for participants who believed they interacted with a human operated (compared to a computer operated) avatar. These results have theoretical as well as practical implications which we will discuss.

## Introduction

Automated deception detection is one of the biggest security challenges for the twenty-first century. More than ever, governments and border agencies are interested in fast, reliable and low-intrusive ways to detect deception in crowded and vulnerable places such as airports (Lu et al., 2010; Damos et al., 2013; Aradau, 2015). The main goal is to quickly filter out those who are low at risk and to flag potential deceit (Derrick et al., 2011; Twyman et al., 2014). A promising approach to meet this goal is to use “automated interview systems” (AIS), which conduct structured interviews and assess deviations in the interviewee's physiology and behavior via sensors (Burgoon and Nunamaker, 2004; Nunamaker et al., 2011; Twyman et al., 2014).

Despite the variety in interfaces, AIS are based on the underlying principle of physiological lie detection; that deception is accompanied by increases in sympathetic nervous system (SNS) activity. This increase is attributed to increased anxiety, negative emotional states, and cognitive demand (Ekman and Friesen, 1969; Zuckerman et al., 1981; Vrij, 2008). Today, AIS are technically advanced enough to sense fluctuations in SNS activity. By sensing and interpreting increases in interviewees' physiological responses, AIS in security contexts have shown promising but mixed results in terms of detecting deception (Derrick et al., 2011; Nunamaker et al., 2011; Twyman et al., 2014). Moreover, we do not know much yet about the underlying factors that makes AIS successful deception detectors.

A critical unanswered question is: Will deceivers' SNS activity increase, knowing they “just” talk to a computer? From interpersonal deception theory (IDT; Buller and Burgoon, 1996) we know that in real face-to-face encounters pressure is put on the deceiver, because deceivers continuously have to manage their expressions in response to suspicion. As a result, emotional stress, cognitive load, and attempted behavioral control increase during deception in interpersonal interactions (Zuckerman et al., 1981). However, these processes are arguably moderated by the type of interpersonal context in which deception takes place (Buller and Burgoon, 1996). The question is whether AIS, operated by computer algorithms, induce similar interpersonal processes in deceivers. Specifically, in a face-to-face encounter, deceivers likely experience fear of getting caught by the interviewer, and put much effort in acting convincingly and therefore often closely monitor themselves and the reactions of the person they deceive (DePaulo et al., 1988; Buller and Burgoon, 1996; Schweitzer et al., 2002; Vrij, 2008). It is questionable whether deceivers exhibit such behavior, knowing their conversation-partner neither is human nor has consciousness. If this is the case, a crucial element for the effectiveness of deception detection with AIS is whether or not deceivers “believe” the system is human or merely computer controlled.

Research demonstrated that people have difficulties to determine whether they interact with a human or a computer controlled entity. For instance, a study based on Milgram's “cyranic illusion” paradigm (Milgram, 1992) showed that participants in a human face-to-face interaction did not notice that their human conversation partners just voiced the words of a computer algorithm in real-time (Corti and Gillespie, 2015a,b). With regards to the content therefore, computers seemingly are capable of being perceived as humans. Moreover, Turing (1950, p. 434) already concluded that when placing the conversation in a context that naturally prevents people to see and touch the “other,” there is “[…] little point in trying to make a ‘thinking machine’ more human by dressing it up […].” Nowadays, such a context can simply be created using a computer-mediated-communication setting (Schroeder, 2011). Lucas et al. (2014) demonstrated for instance that one could let individuals believe that they interacted with a real human or with a computer by simply introducing a virtual avatar as human or computer operated. In fact, an avatar that “looks” and “talks” as a human to convince people might not even be necessary: A study by Morkes et al. (1999) successfully led people to believe that they communicated with a real person in a text-based chat, which actually was a computer algorithm.

Importantly, when providing no clear framing of the “other” as either human or computer operated, people for the identical AIS generally strongly differ in the extent to which they believe they interact with a human or a computer (Schuetzler et al., 2014). The question is whether such perceptions have consequences for how deceivers respond to AIS. There is first evidence that “believing” a virtual avatar is human, opposed to computer, operated indeed alters the behavior toward virtual avatars. A study testing the capability of virtual avatars in clinical screening interviews showed that participants who thought they interacted with a human, disclosed less information and showed higher impression management than participants who perceived the avatar as computer operated (Lucas et al., 2014). According to the authors these results may be explained by an increased feeling that responses are currently judged when indirectly talking to a human. Whereas, in a clinical interview this is disadvantageous, in a deception detection context this may be beneficial.

A higher awareness of being judged would likely help to evoke more cues to deception in a deception detection context. AIS therefore maybe more effective when deceivers perceive such a system as human rather than computer controlled. Indeed, a study by Schuetzler et al. (2014) demonstrated that making an AIS's communication skills more human-like—by building in an adaptive response model—not just increased perceived humanness but also induced faster responses and reduced speech pauses among deceivers. The authors ascribed this to the fact that during deception, deceivers try to maintain their normal pattern of behavior (Buller and Burgoon, 1996). We therefore expected that in order to induce physiological cues to deception, perceiving an AIS as human operated is crucial, and hypothesized that, compared to truth-tellers, deceivers who perceive AIS as computer operated would not differ with respect to SNS activity, whereas deceivers who perceive the AIS as human operated would show an increase in SNS activity.

### The current study

The aim of the current study was to investigate whether perceiving an automated interview system as operated by a human or computer, influences cues to deception in the form of increased SNS activity. Therefore, we conducted an experiment wherein participants were led to believe they participated in a so-called in-basket exercise to test management skills. During the game they were “seduced” to commit fraud by signing a document they were not allowed to. They were then interviewed about this transgression in a structured manner by a human-like avatar on a screen. When confronted with human-like avatars, people generally are uncertain to which degree the avatar really directly represents the actions and thoughts of the person controlling that avatar (Schroeder, 2011). During the interview we measured the participant's electrodermal activity (EDA), the most frequently used measure in the field of physiological lie detection (Vrij, 2000). EDA directly reflects SNS activity and can be measured unobtrusively within one measurement (Wallin, 1981; Dawson et al., 2007; Boucsein, 2012).

In a between-subject design we then let participants deceive the avatar by either lying on all questions or only on the crucial question regarding the signature (which was at the end of the interview). We included the latter condition, because it mirrors real-life deception more closely than when people constantly lie (see Ströfer et al., 2014 for a discussion). Both deception conditions were compared with a condition where participants told the truth. After the interview we assessed whether participants believed the avatar was human or computer operated using a self-report scale.

## Methods

### Participants

We conducted an experiment with 107 graduate students participating in exchange for €5. Because of the limited availability of the computer system running the avatar, and long duration of each experimental session we were somewhat limited in the number of participants we could run. We therefore decided to run as many participants as possible in the available period of 7 weeks. Thirteen participants refused to sign the document that served as basis for the experiment and were excluded from analyses. The data of eight participants who did not follow the instructions of the experiment were excluded as well^1^ and due to technical problems we failed to record EDA data for another seven participants. Therefore, analyses were based on 79^2^ participants (mean age = 21.88, *SD* = 2.84, range = 18–30 years; 41 women). Participants provided written informed consent, and the institutional review board approved the experimental protocol.

### Experimental design and procedure

The experiment consisted of a one-factorial (veracity condition: truth, lie or intention to lie) between-subject design^3^. Participants were randomly assigned to the veracity condition. Participants believed they would take part at a test session for an ostensible newly developed assessment center test (ACT; Sackett and Dreher, 1982). We employed this cover story to allow for deception in a realistic setting. At the start of the experiment we explained to the participants that the ACT consisted of several exercises and that the three best participants completing these exercises each would win 50 €^4^. We further explained that all tasks of the session were relevant for the prize and that we would clearly state when the experiment was finished. On average, the experimental sessions lasted for 1 h. Each session was run by an experiment leader and two confederates: One acting as “experiment assistant” and the other as “interviewer.”

#### In-basket exercise

The experiment started with an in-basket exercise, which often is part of an ACT (Dukerich et al., 1990). Participants were invited to assume the role of a manager of a transport company and to substitute a regular employee who currently was on sick leave. Participants were required to complete four tasks normally executed by the sick employee in 15 min. In the third task participants read a contract that had to be signed by the sick employee—as was indicated by the name of the employee that was already printed on the contract. A note explained that the contract was important for the company and had to be signed urgently. Most of the participants (88%) signed the contract, since this is an easy and fast solvable problem, and continued with the fourth task. However, signing a document under a wrong name is legally not allowed. This transgression served as input for our deception experiment.

#### EDA baseline measurement and confrontation

After finishing the in-basket exercise, participants were brought to the interview room. In order to get an EDA baseline, we attached participants to skin conductance sensors and asked them to sit down for 5 min and relax and wait for the next task of the experiment. We informed participants that this measure assessed the difficulty of the in-basket test. After 5 min the experiment leader entered the room again, stating that she reviewed the participant's output of the in-basket tasks, but that a problem occurred regarding one of the documents. The experiment leader then confronted the participant with the fact that (s)he signed a document (s)he was legally not allowed to sign, and informed that (s)he therefore would be interviewed about this incident by an intelligent virtual agent.

#### Introduction of the virtual avatar

**Figure 1:**
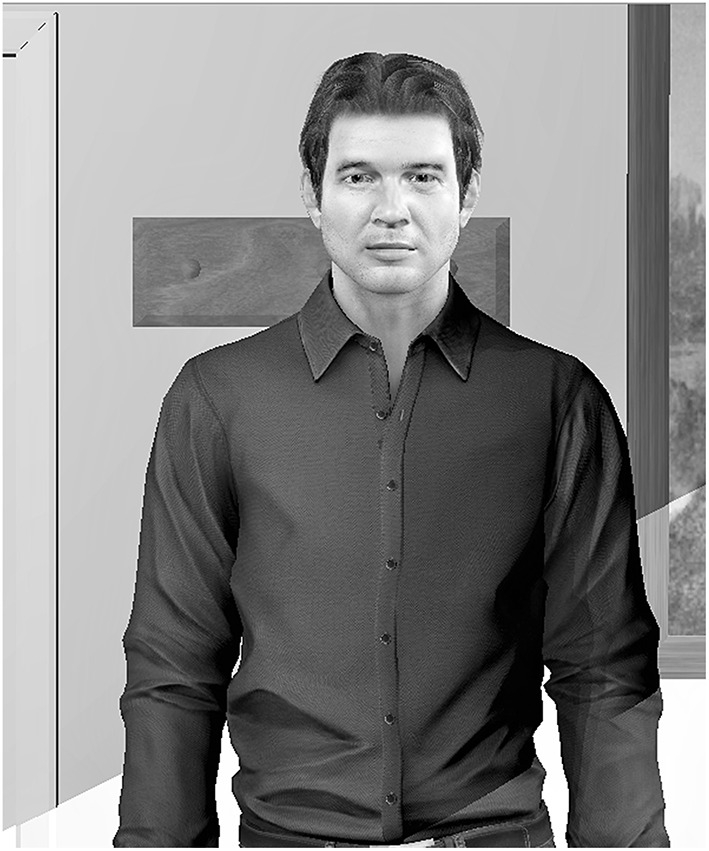
**The avatar (“Brad”) used as a virtual agent in the present experiment (Hartholt et al., 2013)**.

The virtual avatar (see Figure 1) was introduced as an intelligent agent who would appear on the screen (which was turned off during the baseline measurement) in front of the participant. The experiment leader stressed that the virtual agent could hear and see them and that they could talk to him in a normal way. Although in reality the avatar was operated by a human (the experiment confederate), it remained ambiguous to the participants whether or not the avatar acted autonomously or not.

#### Experimental manipulation

After introducing the virtual agent, participants were randomly assigned to a veracity condition. They received a letter advising on the best approach to behave in the upcoming interview about their transgression. This letter formed the experimental manipulation consisting of three veracity conditions: a truth, a lie, and an intention to lie condition. In the *truth* condition, the letter advised participants to tell the truth on all questions, including questions about whether one signed the document. In the *lie* condition, the letter advised to lie on all questions, including questions about whether one signed the document. Finally, in the *intention* condition, the letter advised to tell the truth on all questions but to lie on questions whether one signed the document. We highlighted the question regarding the signature in the truth and lie conditions as well to prevent that the question about the signature would get special meaning in the intention condition only. Hereby we aimed to prevent differences in prospective memory demands between conditions. Finally, we reminded participants that how well they followed the advice would affect their chances for the prize money.

#### The interview with the virtual agent

##### Interview questions

After the participant finished reading the letter the experiment leader turned on the screen and the virtual agent emerged. Then the experiment leader left the room and the interview started. The interview was fully standardized, with the virtual agent asking a total of 10 questions in a fixed order (see Table 1). Each interview started with a number of general questions and worked its way up to the key question revealing the evidence: “Is this your signature on this document?” Thereafter the interview ended with two closing questions. During the interview, participants' EDA was recorded. After the interview the experiment leader entered the room again and asked participants to fill in some final questionnaires, including a short measure assessing the degree to which participants thought the avatar was human or computer controlled.

**Table 1:** **The interview consisted of 10 questions**.

**Question**	**Content**
1	Can you tell about your link with the university? How often and why you are here, what exactly are you doing here?
2	Why did you come to University today?
3	Can you describe step by step what you have done after your arrival?
4	Did you encounter other people? Who?
5	Can you give any other additional information?
6	Did you participate in an assessment center test?
7	In front of you lies a map. Please open it. Have you seen this document before?
8	Is this your signature?
9	Do you want to add something?
10	Was everything clear?

*Questions 9 and 10 were not included in the statistical analyses, since these form the closing part of the interview and were contently not relevant for our experimental manipulation*.

##### Operation of the virtual agent

The virtual agent was portrayed by VHToolkit avatar “Brad” (Hartholt et al., 2013) and appeared on a screen in front of the participant. The virtual agent was operated out of eye shot of the participant from the experiment confederate. The operator of the virtual agent could hear and see the participant on a separate screen and therefore was able to react to the participant's speech in real-time. The operator let the avatar successively ask the 10 interview questions. A new question was selected whenever the participants finished answering the preceding one. In addition, the avatar showed visual and verbal cues of natural listening behavior by for example making short brief utterances such as “M-mh,” “Yes,” and “Ok” while the conversation partner is talking in order to increase the credibility of the virtual agent (Krauss et al., 1977; Heylen et al., 2011). Also, the operator could choose unspecific answers to evade situations in case the participant asks back questions such as “Do you want me to tell more?” The operator could choose out of a set of answers such as “Just go on” (see Table 2).

**Table 2:** **Options the operator used to evade counter questions by the participant**.

**Repertoire of evading answers the operator could give**
Ok
Yes
No
Just go on
Whatever you want
That is for you to decide
Please give an answer

### Measures

#### Perception of virtual agent

We assessed the degree to which participants perceived they interacted with a human or computer with a self-report measure. Our scale was based on the original Turing test (Turing, 1950) in which the test-person interviewed a physically separated real human and an intelligent computer and had to decide which of the two conversation partners is human and which not. In line with more recent studies based on the Turing test, we used a self-report scale assessing the degree to which participants believe they interacted with a real human or a computer (Person et al., 2002; Ijaz et al., 2011; Schuetzler et al., 2014). Participants completed five items such as “I think the virtual agent was controlled by […]” or “I think the questions were selected by […]” on a 7-point Likert scales ranging from 1 (*A human*) to 7 (*A computer*), Cronbach's alpha = 0.80. A factor analysis on these five items (method: maximum likelihood, based on Eigenvalues greater than 1) revealed one single underlying factor, explaining 56.02% of the variance, corroborating the intention to measure one construct: the believe in whether an Avatar is human or computer controlled. We therefore created an overall score by computing the mean value for the scores on the five items (Distribution of scores across participant sample: *Mdn* = 3.40, *M* = 3.52, *SD* = 1.59, Range: 1–7).

#### Electrodermal activity (EDA)

##### Recording

EDA was recorded continuously with 256 Hz from baseline till the end of the interview (down-sampled to 16 Hz during off-line analysis). EDA was recorded exodermal (constant voltage) via skin conductance using skin conductance sensors (Thought Technology Ltd., Montreal West, Quebec, Canada), attached to the distal phalanx of the right index and ring fingers (Boucsein, 2012). The signal was amplified using ProCompInifiniti amplifier (Thought Technology Ltd.) and was recorded in μS.

##### Analyzing

To assess physiological arousal during the interview, we executed a Continuous Decomposition Analysis using Ledalab (Benedek and Kaernbach, 2010) which is an algorithm written in MATLAB. We focused on tonic EDA which describes the overall conductivity of the skin over longer time intervals and can be operationalized by the skin conductance level (SCL; Figner and Murphy, 2010). To assess tonic EDA during the interview, we subtracted the SCL during the interview from the SCL during the baseline measurement^5^. As recommended by Boucsein (2012), EDA was normalized by taking the natural logarithm. Statistical analyses were performed on log-transformed data, but the reported descriptive statistics were based on the raw data (in μS).

## Results

Demographics across conditions were equally divided between veracity conditions and therefore were not included in further analyses. To test the hypotheses, we conducted a multiple regression to test whether perceiving the avatar as human operated, increases cues to deception in form of tonic EDA (see Table 3). All data assumptions for linear models were met (Field, 2013). We ran a regression model with the standardized scores of avatar perception, two dummy variables dummy coding the experimental conditions, and two interaction terms as predictors. For the dummy variables we used the truth condition as reference condition. Dummy 1 compared the lie condition (lying = 1, intention = 0, and truth = 0) and Dummy 2 the intention condition (lying = 0, intention = 1, truth = 0) to the truth condition.

**Table 3:** **Multiple regression table: Regression Beta's predicting EDA**.

**Predictor**	**Model 1**		**Model 2**	
	**β**	***p***	**β**	***p***
Dummy 1	0.37	0.029	0.37	0.022
Dummy 2	0.30	0.104	0.32	0.068
Avatar perception (z-score)	0.01	0.873	0.23	0.033
Dummy 1 × Avatar perception			−0.46	0.007
Dummy 2 × Avatar perception			−0.30	0.086

*High scores on the Avatar perception scale indicate that participants believed to interact with a computer rather than a human. Dummy 1 contrasts the lie to the truth condition and Dummy 2 the intention to the truth condition*.

**Figure 2:**
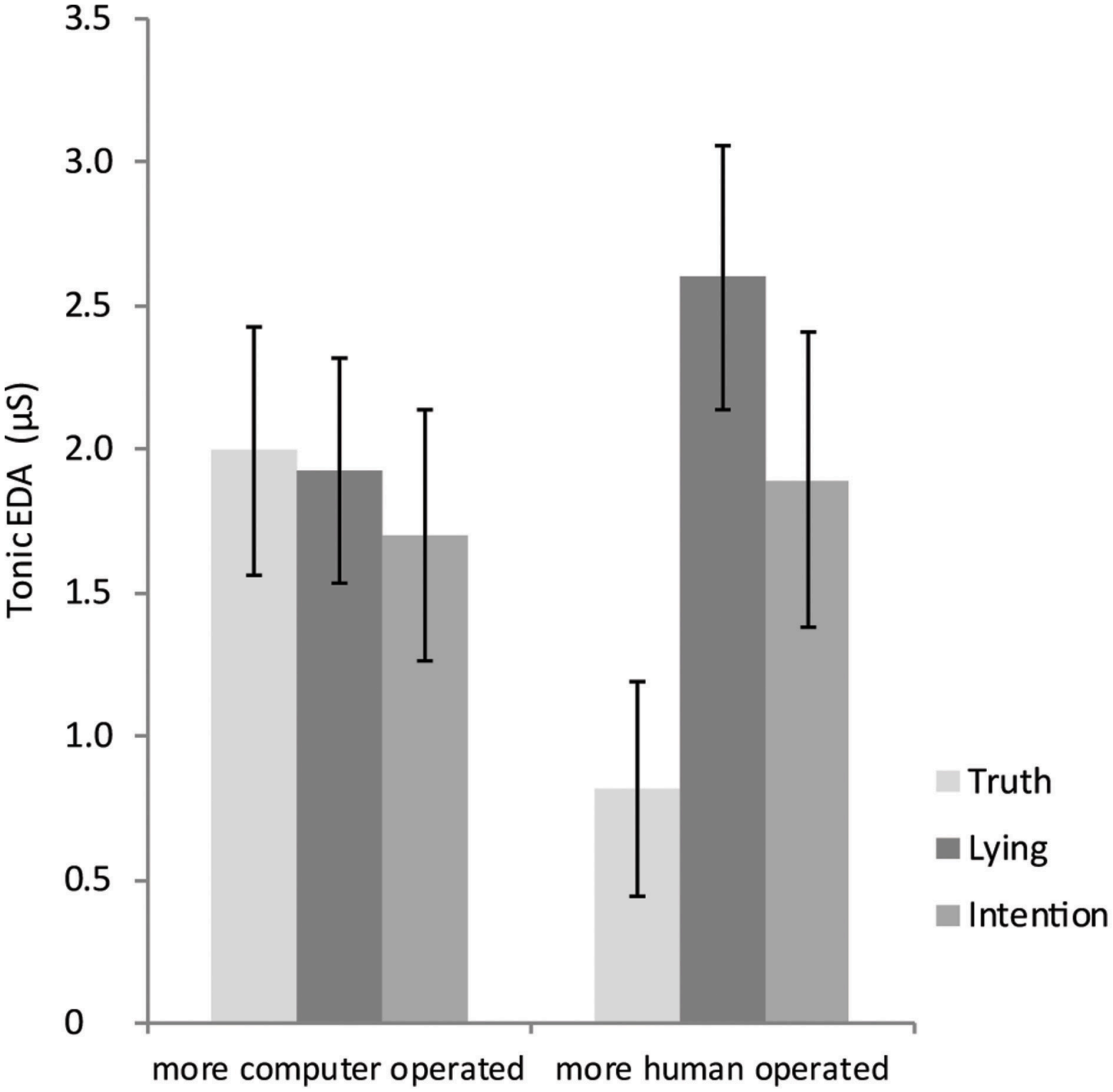
**The interaction effect between condition and the perceived operation of the avatar on tonic EDA**. The bars with standard error present the predicted tonic EDA value for people who score 1 *SD* from the mean toward the computer anchor and 1 *SD* toward the human anchor on the perceived avatar operation scale within the three veracity conditions.

In line with our hypothesis, we found a significant effect of Dummy 1, β = 0.37, *t*_(71)_ = 2.34, *p* = 0.023, revealing that SCL was higher in the lie (*M* = 2.29, *SE* = 0.28) compared to truth condition (*M* = 1.47, *SE* = 0.29). This effect was qualified by a significant and medium (Cohen, 1988) interaction effect of Dummy 1 and avatar perceptions, β = −0.46, *t*_(71)_ = −2.77, *p* = 0.007. Simple slopes analyses (Aiken et al., 1991) revealed that for participants who perceived the avatar as relatively human operated (1 *SD* from mean toward human anchor) SCL was significantly higher in the lie than in the truth condition, β = 0.83, *t*_(71)_ = 3.63, *p* = 0.001. However, for participants perceiving the avatar as relatively computer operated (1 *SD* from mean toward computer anchor), the difference in SCL between the lie and truth condition disappeared, β = −0.08, *t*_(71)_ = −0.35, *p* = 0.726 (see Figure 2).

A similar pattern was found when comparing the intention with the truth condition. Although the main effect of Dummy 2 was not significant, β = 0.32, *t*_(71)_ = 1.85, *p* = 0.068, the effect size indicates a medium effect in the predicted direction, showing that SCL was higher in the intention (*M* = 1.80, *SE* = 0.32) compared to the truth condition (*M* = 1.47, *SE* = 0.29). The interaction effect of Dummy 2 with perceptions of the avatar was not significant, β = −0.30, *t*_(71)_ = −1.74, *p* = 0.086 (see Figure 2). But also here the effect size indicates an, albeit medium, effect in the predicted direction. That is, simple slopes analyses showed that for participants who perceived the avatar as more human operated (1 *SD* from mean toward human anchor) the difference in SCL between the intention and truth condition was significant, β = 0.62, *t*_(71)_ = 2.58, *p* = 0.012. However, for participants who perceived the avatar as computer operated (1 *SD* from mean toward computer anchor) there was no significant difference in SCL between the intention and truth condition, β = 0.02, *t*_(71)_ = 0.10, *p* = 0.924. As expected, the tonic EDA differences between the truth and respectively the lie and intention condition thus became larger when participants believed the avatar would be human instead of computer operated.

We also examined the condition by perceptions interaction effects by conducting a simple slopes analysis of avatar perceptions within the veracity conditions. This analysis enables us to see whether there are EDA difference within each veracity condition depending on whether participants perceived the avatar as human or computer-controlled. When participants believed the AIS was computer instead of human operated, simple slopes analyses within each veracity condition revealed that in the truth condition EDA significantly increased, β = 0.23, *t*_(71)_ = 2.18, *p* = 0.033, whereas in the lie condition, tonic EDA seem to remain the same, β = −0.22, *t*_(71)_ = −1.77, *p* = 0.080. However, a small effect size revealed a trend that participants EDA in the lie condition decreased as they believed the AIS was computer instead of human operated. In the intention condition, EDA was not influenced by the degree to which participants believed they interacted with a computer, β = −0.06, *t*_(71)_ = −0.48, *p* = 0.633.

## Discussion

Automated physiological lie detection may work—but the results of our study suggest that it is crucial that deceivers believe that the system they are interacting with is controlled by another human and not by an intelligent computer. In the current study we examined deceivers who interacted with a virtual avatar about whom they were uncertain whether it was human or computer operated. We found that the degree to which participants perceived they interacted with a human controlled avatar intensified cues to deception in the form of heightened SCLs, a common marker for psychophysiological lie detection (Vrij, 2008). In line with our hypothesis, EDA differences between deceivers and truth tellers became significantly more pronounced when participants believed they interacted with a human operated avatar. When participants perceived the avatar as computer operated, there was no difference between truth tellers and deceivers. There was a medium effect suggesting that this difference in EDA between deceivers and truth-tellers seem to be still present, even when deceivers only lied on one—crucial—question regarding the transgression. This last finding has important practical significance, because in reality deceivers tend to tell the truth as much as possible and only lie on crucial parts in a conversation (Strömwall et al., 2006; Leins et al., 2013).

Moreover, the present results showed that both deceivers as well as truth-tellers may be affected by their perceptions of AIS. That is, as predicted, part of the decline in EDA differences between lying and truth telling when the avatar was perceived as more computer than human-operated, could be explained by a trend, showing a *decrease* in EDA when people perceived the avatar as being computer (vs. human) operated and were lying. Additionally, we also—unexpectedly—found that EDA *increased* when people perceived the avatar as computer instead of human-operated and were telling the truth.

Based on IDT (Buller and Burgoon, 1996), we indeed predicted that a virtual avatar which is believed to be computer-operated might not elicit the typical psychological responses (such as an increase in monitoring processes) typical for deceivers compared to truth-tellers. In interpersonal interactions perceivers usually believe their lies shine through and therefore experience a greater sense of awareness with regard to their performance than truth tellers (Gilovich et al., 1998; Elaad, 2003; Vrij, 2008). Despite the technical abilities of current AI systems to conduct an interview and to sense physiological arousal, today's AIS however just “pretend to act like” humans instead of being one. AIS, for instance, have no consciousness in terms of noticing the presence of themselves in the world (Khanna et al., 2015), which may have important consequences for how people socially respond in interactions with AIS. For example, a study by Schuetzler et al. (2014) demonstrated that deceivers exhibit shorter response latencies and pause lengths when deceiving a chat bot which was perceived as more human-operated compared to a chat bot that was perceived as computer-operated. The authors explained this by the fact that deceivers felt a greater sense of urgency to respond quickly due to their desire to appear truthful when interacting with a human-operated system.

Similarly, deceivers who assume to talk to an unconscious computer which does not “understand” the conversation on a social level may feel less pressure to monitor the course of the deceptive conversation on a meta-level such as they would do when talking to a real human (Buller and Burgoon, 1996). Consequently, deceivers may be less engaged in monitoring behavior and feel no need to maintain a normal (unsuspicious) conversation while deceiving. Perceiving an AIS as computer rather than human-operated thus indeed should result in less demanding cognitive and affective processes and, consequently, lower EDA normally associated with deception (Spence et al., 2001; Vrij, 2008).

In addition, the present results revealed that truth tellers showed an opposite pattern: EDA increased to the extent to which truth-tellers believed the avatar was operated by a computer and decreased to the extent to which they believed the AIS was operated by a human. This result implies that also for truth-tellers' physiological reactions perceiving an AIS as a conscious social being vs. a socially inapt computer may be crucial—and specifically so in the current setting. That is, truth tellers generally believe that their innocence automatically shines through (Gilovich et al., 1998; Vrij, 2008). In the present study truth tellers were in fact not innocent, because they did put an unauthorized signature on the document. Participants however did not commit the transgression on purpose, and may see it as minor transgression which would not be a big problem to admit when explained. It may be possible that truth tellers might have felt that an AIS controlled by a socially aware human would understand their good intentions, but that a computer directed system would completely miss this aspect of their transgression, and would judge them strictly on the basis of their actions.

### Recommendations and limitations

With the rise of new technology, the findings of the current study not only have theoretical but also practical relevance. From a theoretical view, it supplements the IDT by Buller and Burgoon (1996) who emphasize the importance of interpersonal contact and interactivity in communication for cues to deception. We supplement these factors by adding “believing” to communicate with a real human being as a third crucial factor for cues to deception. When using an AIS in a physiological lie detection context, it may be important to indicate to interviewees that the avatar is human controlled—as this can help to better discriminate between honest and deceitful persons.

The ultimate aim of a AIS in a security context is to detect deception in high-stake contexts such as border control—where the stakes for the deceiver often are high—and consequences of failing to detect deceit severe. Unlike previous studies, where participants are explicitly instructed to commit something unlawful (e.g., Kircher et al., 1988; Verschuere et al., 2004; Gödert et al., 2005), we therefore used a paradigm which maximize ecological validity. Participants were “seduced” to commit a transgression and were subsequently advised how to respond when being interviewed. This should increase the participant's agency and increase the stakes (DePaulo et al., 2003; Sip et al., 2008). Research on the behavioral correlates of deception show that particularly high-stakes lies are more difficult for the deceivers and associated with intense emotions (Porter and Brinke, 2010). Corroborating the high-stakes nature of the current paradigm there were more participants having difficulties following the deception instructions (in the lie as well in the intention condition) than participants following the truth-telling instructions. However, this also resulted in more participants being excluded from the lie and intentions conditions than the truth condition (see Footnote 1), which may have caused a bias in participant selection. The consequences for the current results of this difference in drop-outs should be limited because, the stress and cognitive demand these participants were experiencing when attempting to follow the instructions should increase EDA. Thus, including these subjects in the analyses (if this would have been possible), if anything would have increased the mean EDA in the lie and intention conditions, making the differences between the truth vs. the lie and intention condition even more pronounced.

Moreover, we found small effects, suggesting that also truth tellers may be differentiated from deceivers who only lied on one crucial question and for the rest of the interview stuck to the truth. Similar to the lie condition, it seems however crucial that the interviewees believed that a human operated the virtual interviewer. This finding is of great practical relevance as real-life deceptive attempts typically consist for a great part of truth telling (with the intention to lie) and only a few literal lies (Carlson et al., 2004; Strömwall and Willén, 2011; Leins et al., 2013). The reason for this is twofold: Staying close to truth is easier and costs less effort (Leins et al., 2013), and it reduces chances of being caught by for instance delivering contradicting information (Hartwig et al., 2010).

The current as well as previous work (e.g., Schuetzler et al., 2014) show that people naturally differ in the extent to which they perceive an AIS as being human controlled or an autonomously computer controlled system. Having established that the “mere belief” to communicate with a real human is crucial, we also recommend manipulating experimentally the actual operation of the avatar and belief of how the avatar is operated (see for example Lucas et al., 2014). Another interesting question for future research is why people differ in the perceptions of such systems. This may be correlated with certain personality traits such as emotional stability (e.g., Vries et al., 2009), or for instance tied to the level of experience someone has with how computer systems operate.

Our findings also have broader implications for the field of deception detection. Deception is defined as “creating in another a belief which oneself considers to be untrue” (Vrij, 2008). Deception thus by definition is an interpersonal activity, including a deceiver and target to deceive. However, a lot of physiological deception studies neglect that deception takes place within a context of social interaction and study deception in isolation. By placing the emphasis on a clean and controlled study environment, it often is neglected that deception by definition is socially rooted (Sip et al., 2008). The deceptive activity however—whether it is literary lying, bluffing or omitting facts (Carlson et al., 2004)—by its own nature is associated with “mentalizing”—“the ability to predict and explain the mental states of other people […]” (Grezes et al., 2006). Deceivers need to keep track what the target to deceive knows and thinks to anticipate the deceptive course of action (Sip et al., 2008). Therefore, the target to deceive plays a crucial role for the psychological processes underlying deception. Hence, it should be beneficial for detecting cues to deception to let the suspect communicate with—or at least believe to communicate with—a real human being.

## Conclusion

One of the challenges of the twenty-first century is automated deception detection. The current study discovered a crucial ingredient that makes such systems work—the belief that one is talking to a human instead of an autonomous computer-operated system. We advise research and security industry to take this into account when putting the results of experimental studies into real-life applications such as an automated interview system for deception detection. Already existing AIS are technically sophisticated and even “look” human-like by means of an avatar interface (Derrick et al., 2011; Nunamaker et al., 2011). The crux of matter however may be not that these systems look human-like, but that they are perceived as human-operated. In other words, the suspects have to have the belief that they talk to a conscious human through the virtual avatar and not a machine.

## Author contributions

Conceived and designed the experiments: SS, EG, EU, and MB. Performed the experiments: SS, MB. Analyzed the data: SS, EU. Contributed reagents/materials/analysis tools: MN, MB. Wrote the paper: SS EU, MN, EG.

### Conflict of interest statement

The authors declare that the research was conducted in the absence of any commercial or financial relationships that could be construed as a potential conflict of interest.

## Footnotes

^1^One participant failed to follow the instructions in the truth, four participants in the lie, and three in the intention condition. The subject in the truth condition committed a technical “fault,” one participant in respectively the lie and intention condition refused to follow the instruction, and the other participants ostensibly were so demanded/stressed that they did not manage to follow the instructions (e.g., constantly lie).

^2^The 28 of 107 participants who had to be excluded were randomly divided across conditions.

^3^The original design also contained interview style (information-gathering vs. accusatory style) as an additional between-subject factor. Analyses with this factor did not render any significant effects and therefore was eliminated from further analyses.

^4^In reality, the money was allotted among the participants after the experiment.

^5^We verified the results by also using another way of calculating tonic EDA. Employing CDA analysis by Benedek and Kaernbach (2010), we used the average phasic driver activity across the interview as dependent variable in the regression model. This estimates the phasic activity of the electrodermal activity which overlays the skin conductance level (Figner and Murphy, 2010). Similar significant patterns were found using the phasic driver activity as using the SCL.
